# Functions and challenges of a government-run animal bite treatment center in the delivery of rabies post-exposure prophylaxis services in Iloilo City, Philippines: A 6-year descriptive study, 2018–2023

**DOI:** 10.1016/j.dialog.2025.100266

**Published:** 2025-12-11

**Authors:** Anna-Lee B. Bandoy, Neil Marc S. Dasas, Alpha Issa Christianne P. Abegonia, John Piox J. Badiang, Chester Lloyd S. Berdan, Maria Lucille M. Magallanes, Jes Ivan D. Sian, Therese A. Sumague, Patricia Kei P. Tulio, Addy Mae P. Binoya, Ma. Cristina L. Erum, Alfredo A. Hinay

**Affiliations:** a College of Medicine, West Visayas State University, La Paz, Iloilo City, Philippines; b Graduate School Department, University of the Immaculate Conception, Davao City, Philippines

**Keywords:** Public health, Philippines, Animal bite treatment center, Rabies, Post-exposure prophylaxis

## Abstract

**Background:**

Animal bite injuries are a serious public health concern due to the risk of rabies infection. In March 2024, the Department of Health (DOH) reported 84 rabies cases with six fatalities from Iloilo City. The most crucial management of animal bite injuries includes immediate wound care and rabies post-exposure prophylaxis (PEP), typically administered at an Animal Bite Treatment Center (ABTC). In 2007, the Philippines established the Republic Act No. 9482, also known as the Anti-Rabies Act, which created the National Rabies Prevention and Control Program (NRPCP) to control and eliminate rabies. This study aimed to describe the functions and challenges faced by one of these government-run ABTCs in Iloilo City, specifically a) provision of rabies PEP for animal bite cases, b) instructions for proper wound care, c) documentation of animal bite cases, and d) conduct of health promotion activities.

**Materials and methods:**

This mixed-methods study was conducted at an ABTC in Iloilo City between January and March 2024. The first phase of the study collected secondary data from the official registry from January 1, 2018, to December 31, 2023. In the second phase, data were collected through direct observation of practices during a site visit in February 2024. A validated checklist based on the DOH Manual of Operations and WHO health system framework was developed and used for objective points of observation. Descriptive analyses of frequencies and percentages were conducted using Microsoft Excel and compared with the NRPCP guidelines. Also, Mann-Kendall test was conducted to evaluate the temporal trends in bite incidence proportions.

**Results:**

The non-hospital-based government ABTC operated under the Iloilo City Health Office provided a.) rabies PEP and wound care for animal bite cases, b.) documentation of animal bite cases, and c.) awareness campaigns. A total of 20,134 animal bite cases were documented from 2018 to 2023. Three types of vaccines were delivered to the center: Purified Vero Cell Rabies (PVRV) and Purified Chicken Embryo Cell (PCEC) for active immunization, and Equine Rabies Immunoglobulin (ERIG) for passive immunization. In 2023, the lowest number of category III ERIG recipients was 42.65 % (*n* = 534). Despite COVID-19 restrictions from 2020 to the middle of 2022, all patients completed their TCV vaccination between 2020 and 2023. Challenges, such as vaccine shortages, record inconsistencies, and referral issues, persist.

**Conclusion:**

The non-hospital-based government-run ABTC has maintained rabies PEP services in Iloilo City from 2018 to 2023, despite challenges. Collaboration with the Local Government Unit (LGU) and DOH, increased campaigns, and lay lectures on the prevention of animal bites and rabies infection, along with increased healthcare funding, are needed for sustainable solutions.

## 1 Introduction

Animal bite injury is a serious public health concern due to its risk for rabies infection, which if left untreated, is almost always fatal [[1], [2], [3], [4]]. The Department of Health (DOH) reported 84 rabies-related fatalities between January and March 16, 2024, with Iloilo City accounting for six deaths [[5], [6], [7]]. Effective management of animal bite injuries includes immediate wound care and rabies post-exposure prophylaxis (PEP), typically administered at an Animal Bite Treatment Center (ABTC) [[1], [2], [3],^8^,^9^]. These centers provide free access to anti-rabies vaccines (ARV) and rabies immunoglobulin (RIG) based on the World Health Organization (WHO) bite categorization guidelines [2,[10], [11], [12]].

To provide better access to PEPs, the Philippines has expanded its network of Animal Bite Treatment Centers (ABTCs) which offers free access to WHO pre-qualified human ARV and RIG [[7], [8], [9]]. Since 2007, the Philippines has implemented Republic Act No. 9482, or the Anti-Rabies Act, which established the National Rabies Prevention and Control Program (NRPCP) to combat and hopefully eradicate rabies [[9], [10], [11],^13^,^14^]. As of 2024, Iloilo City has two active government-run ABTCs — one hospital-based and one community-based [11].

With the rising demand for PEP and the global effort to eliminate human rabies, ABTCs must continuously enhance their capacity to meet these growing needs [14,15]. One of the biggest challenges seen is the lack of support and implementation of RA 9482 due to local executives disregarding the impact of rabies [6,11]. The study by Amparo et al. (2018) mentions several problems that the centers and the system face, including the lack of vaccines supplied by the national government, a shortage of trained staff, a lack of continuous learning among staff, and delayed implementation of updated guidelines [3,6,9]. To support the expansion of anti-rabies initiatives, existing programs and institutions should be reviewed for their key implementation functions, and for all challenges they have been encountering in their locale. This study would like to describe the functions and challenges of the only non-hospital based government-run ABTC in Iloilo City, specifically a.) rabies PEP for animal bite cases, b.) instructions for proper wound care, c.) documentation of the animal bite cases, and d.) conduct of health promotion activities.

## 2 Materials and methods

### 2.1 Study design

**Fig. 1:**
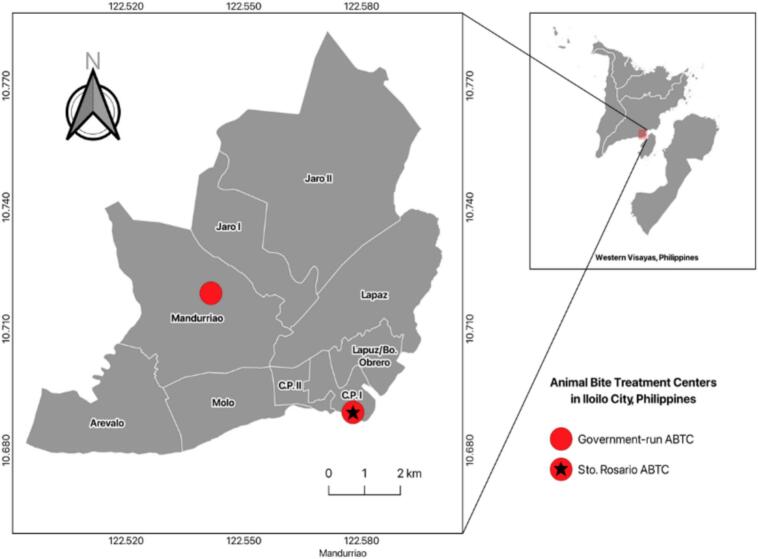
Location of animal bite treatment centers in Iloilo City, Philippines. Quantum GIS (QGIS) v3.36 software.

This mixed-methods observational study was conducted from January 1, 2018, to March 31, 2024, at a government-run ABTC in Iloilo City, Philippines. The ABTC operates under Sto. The Rosario District Health Center and Iloilo City Health Office provide rabies post-exposure prophylaxis (PEP), wound care, and documentation services to patients from nine city districts (Fig. 1). Ethical clearance was obtained from the West Visayas State University College of Medicine Research Office and Unified Research Ethics Review Committee (URERC).

### 2.2 Setting

The study population comprised all patients with animal bites recorded at the ABTC between January 1, 2018, and December 31, 2023. The exclusion criteria were data unrelated to human patients, high-risk populations outside the typical catchment area, and individuals who received pre-exposure prophylaxis (PrEP). Patients with incomplete or reliable records were excluded to ensure accurate data.

### 2.3 Variables

The primary variables included the annually reported animal bite incidence, patient demographics (age and sex), bite source/type, rabies exposure categories (I, II, and III), vaccination and immunoglobulin administration status, mortality, and district of residence. Data were sourced from the official registry maintained by the ABTC and complemented by direct on-site observations in February 2024. To assess center functions, a validated checklist adapted from the Department of Health Manual of Operations and the WHO Health Systems Framework was used. These variables were selected based on the established epidemiological reporting standards of the Department of Health and the WHO, ensuring comparability with national surveillance indicators. Parameter definitions and classifications were cross-validated against the DOH surveillance manual to maintain consistency and reproducibility.

### 2.4 Data collection

The study was conducted in two phases to ensure comprehensive data acquisition and robust evaluation of the ABTC operations. In the first phase, retrospective secondary data were extracted from the official ABTC registry from January 1, 2018, to December 31, 2023. This registry provides detailed records of animal bite cases, including annual incidence rates, demographic profiles, mortality outcomes, vaccine utilization, and administration of Equine Rabies Immunoglobulin (ERIG). Specific data points included cumulative and district-level case distributions, the number of new cases managed annually, and the number of complete and incomplete Tissue Culture Vaccine (TCV) courses.

The second phase involved a site visit conducted in February 2024, during which the current center practices were directly observed and systematically assessed. A structured checklist developed by the research team and grounded in the Department of Health (DOH) Manual of Operations, the World Health Organization (WHO) Health Systems Framework, and extant best-practice literature was used to evaluate six critical functional domains: (1) service delivery and facilities, (2) health workforce, (3) health information systems, (4) access to essential medicines, (5) health financing, and (6) leadership.

Data extraction and observational assessments were conducted using standardized protocols to maximize data integrity and mitigate potential sources of bias. The vaccination and referral procedures strictly adhered to the prevailing DOH and WHO guidelines. The completeness and accuracy of the secondary data relied on the routine documentation practices maintained by the ABTC. Because this study used only secondary data, no direct patient interaction or consent was required. All extracted information was de-identified prior to the analysis, and no personally identifiable data were included in the analysis. For the site visit and observational phase, informed consent was obtained from the participating ABTC staff before observation and data collection. All collected data were securely stored and accessible only to the research team in accordance with the approved protocols of the West Visayas State University College of Medicine Research Office and the Unified Research Ethics Review Committee (URERC).

### 2.5 Inclusion and exclusion criteria

The study focused on data collected from the ABTC covering the period from January 1, 2018, to December 31, 2023. The researchers relied solely on the available recorded data maintained by the ABTC, making the completeness and accuracy of the study reliant on the effectiveness of the center's data recording practices. The researchers also based their data on the directly observed practices of the center at the time of site visits. Practices with no written protocols in place and no physical proof were not included in the study. Patients with incomplete or missing essential information (such as sex, age, bite source, or exposure category) were excluded from the analysis to maintain data quality and consistency. The inclusion and exclusion decisions were independently verified by two researchers to ensure consistency, and discrepancies were resolved through discussion to minimize subjective bias in data selection.

### 2.6 Bias

Potential sources of bias include reliance on secondary data from the ABTC registry, which may have introduced errors due to incomplete, inconsistent, or inaccurate documentation of the data. Additionally, the use of routine registry data could result in under-reporting or missing data. Observational assessments were limited to recording procedures, potentially introducing information bias by excluding undocumented activities from the analysis. To minimize these biases, data triangulation was employed by comparing the registry data with the field observations and interview findings. Validated tools and standardized data collection checklists were used to enhance the reliability and consistency.

### 2.7 Statistical analysis

Bite incidence data from the Animal Bite Treatment Center in Iloilo City spanning 2018–2023 were analyzed by sex, age group, bite source, and rabies exposure category. The yearly counts and proportions were calculated for each variable. Differences in the distribution of bite incidents across variables were evaluated using the chi-square test. To assess temporal trends in the bite incidence proportions for the overall Iloilo City population, the Mann-Kendall test for monotonic trends was applied separately by variable. This nonparametric test was chosen because it does not require assumptions of normality or linearity and is well-suited for detecting consistent increasing or decreasing trends in annual proportions, particularly with relatively small sample sizes typical of public health surveillance data. The test treats years as ordinal variables to evaluate the statistically significant monotonic trends over time. Statistical significance was set at *p* < 0.05. All analyses were performed using Stata 14 (StataCorp LP).

## 3 Results

### 3.1 Epidemiology and trends of animal bite incidents in Iloilo City

Between 2018 and 2023, the Animal Bite Treatment Center (ABTC) recorded 20,134 animal-bite cases (Table 1). Females represented a statistically significant majority of bite victims (53.48 %) compared with males (46.52 %) (*p* < 0.001). Age distribution analysis revealed that 67.98 % of the cases occurred in individuals older than 15 years, significantly exceeding the 32.02 % incidence among younger patients (*p* < 0.005). Dog bites were the most prevalent source of injury, significantly surpassing bites from other animals (*p* < 0.001). Rabies exposure was predominantly classified as Category II, accounting for 72.12 % of cases, while Category III exposure accounted for 27.88 % (*p* < 0.001).

**Table 1:** Distribution of bite incidents in Iloilo City, 2018–2023.

	n (%)	*p value*
Sex		
Male	9351 (46.52)	<0.001
Female	10,752 (53.48)	
Age		
<15 years old	6447 (32.02)	0.005
>15 years old	13,685 (67.98)	
Bite source		
Dog	11,954 (60.06)	<0.001
Cat	7923 (39.81)	
Others	26 (0.13)	
Rabies exposure category		
Category II	14,521 (72.12)	<0.001
Category III	5613 (27.88)	

Note: Discrepancy in the total is due to incomplete entries in the rabies exposure registry of the center.

**Fig. 2:**
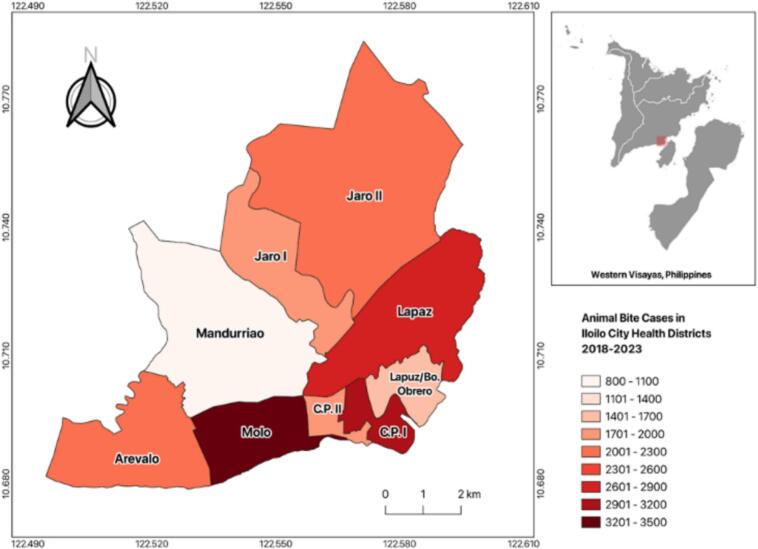
Cumulative distribution of animal bite cases treated from 2018 to 2023. Quantum GIS (QGIS) v3.36 software.

**Fig. 3:**
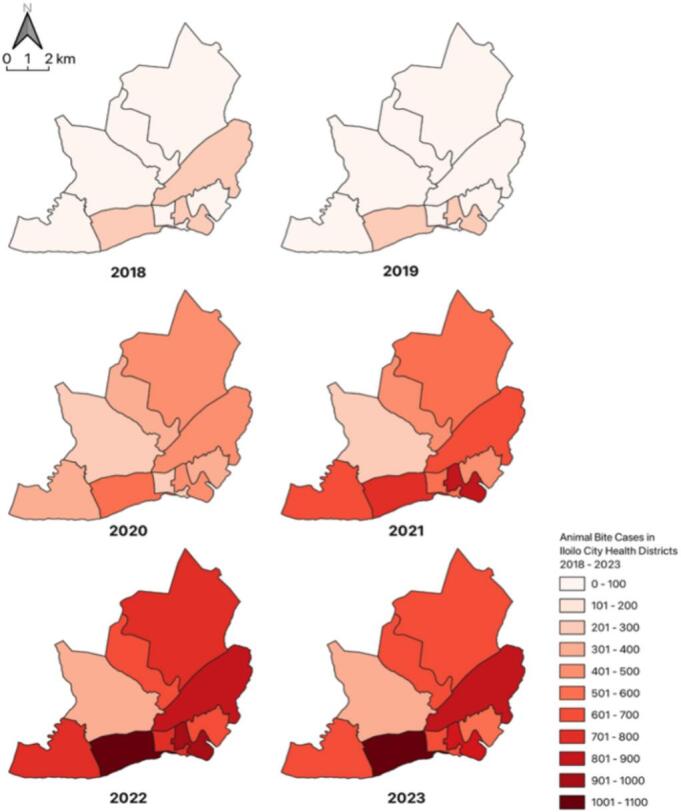
Annual distribution of animal bite cases per district treated from 2018 to 2023. Quantum GIS (QGIS) v3.36 software.

Spatial analysis further demonstrated variations in case distribution across city districts. Fig. 2 illustrates the cumulative distribution of animal bite cases treated per district from 2018 to 2023, with Molo District reporting the highest number of cases (*n* = 3515) and Mandurriao the lowest (*n* = 879). The annual distribution of animal bite cases per district is shown in Fig. 3, highlighting temporal fluctuations and geographic disparities across the service area.

Despite observing statistically significant differences among demographic and exposure variables, trend analysis revealed no significant monotonic increase or decrease in bite incidence proportions over the study period (2018–2023) (Table 2). Specifically, the temporal trends by sex, age group, and bite source showed no significant change (*p* = 0.0602). In contrast, rabies exposure categories revealed a significant monotonic trend for Category II (*p* = 0.0241), whereas Category III showed stability over time (*p* = 0.1328).

**Table 2:** Distribution and trend analysis of bite incidents by demographic and exposure characteristics in Iloilo City, 2018–2023.

	Overall n (%)	2018 n (%)	2019 n (%)	2020 n (%)	2021 n (%)	2022 n (%)	2023 n (%)	*p-value for trend*
Sex								
Male	9351 (46.52)	432 (4.62)	354 (3.79)	1246 (13.32)	1945 (20.80)	2912 (31.14)	2462 (26.33)	0.0602
Female	10,752 (53.48)	474 (4.41)	366 (3.40)	1341 (12.47)	2233 (20.77)	3360 (31.25)	2978 (27.70)	0.0602
Age								
<15 years old	6447 (32.02)	262 (4.06)	186 (2.89)	837 (12.98)	1473 (22.85)	2087 (32.37)	1602 (24.85)	0.0602
>15 years old	13,685 (67.98)	644 (4.71)	490 (3.58)	1723 (12.59)	2805 (20.50)	4185 (30.58)	3838 (28.05)	0.0602
Bite source								
Dog	11,954 (60.06)	547 (4.58)	425 (3.56)	1561 (13.06)	2754 (23.04)	3868 (32.36)	2799 (23.41)	0.0602
Cat	7923 (39.80)	356 (4.49)	293 (3.70)	1023 (12.91)	1519 (19.17)	2394 (30.22)	2338 (29.51)	0.0602
Others	26 (0.14)	3 (11.54)	2 (7.69)	3 (11.54)	5 (19.23)	10 (38.46)	3 (11.54)	0.3140
Rabies exposure category								
Category II	14,521 (72.12)	459 (3.16)	538 (3.70)	1979 (13.63)	2730 (18.80)	4617 (31.80)	4198 (28.91)	**0.0241**
Category III	5613 (27.88)	445 (7.93)	115 (2.05)	608 (10.83)	1548 (27.58)	1655 (29.49)	1242 (22.13)	0.1328

### 3.2 Mortality

From 2018 to 2020, no rabies-related mortality was reported in the districts of Iloilo City. However, mortality events have emerged in subsequent years, with one death recorded in 2021 and 2022. Notably, there was an increase in 2023, with three fatalities reported; one case originated from the district of Arevalo and the remaining two were from Molo.

### 3.3 Vaccines delivered

**Fig. 4:**
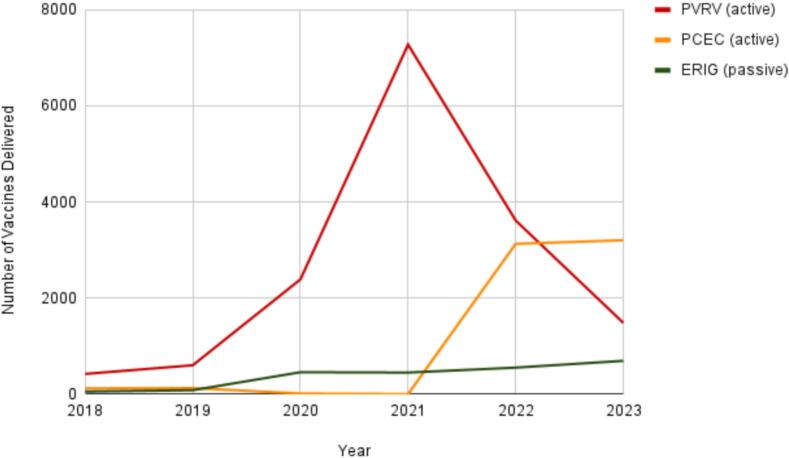
Number of vaccines delivered to the government-run ABTC from 2018 to 2023.

The ABTC has administered three main types of immunobiologicals for rabies prevention: Purified Vero Cell Rabies Vaccine (PVRV) and Purified Chicken Embryo Cell vaccine (PCEC), collectively known as Tissue Culture Vaccines (TCV), for active immunization, along with Equine Rabies Immunoglobulin (ERIG) for passive immunization. Between 2018 and 2021, PVRV usage increased exponentially, reflecting the enhanced vaccine availability and increased demand. Conversely, the PCEC distribution remains relatively low until 2022, indicating limited adoption or constrained supply. Throughout the study period, ERIG administration remained consistently low, highlighting the potential limitations in the access or utilization of passive immunization (Fig. 4).

**Fig. 5:**
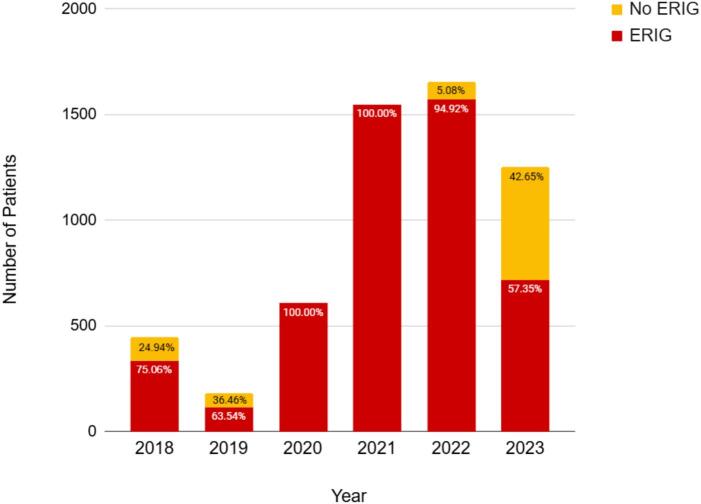
Number of category III patients who received ERIG from 2018 to 2023.

Categories of rabies exposure are critical for guiding appropriate post-exposure prophylaxis (PEP). Fig. 5 shows the number of Category III patients who received ERIG, highlighting an important decline in 2023, with only 42.65 % (*n* = 534) of the highest-risk group receiving ERIG.   This aligns with the vaccine delivery patterns shown in Fig. 4 where PVRV, PCEC, and ERIG were administered at the Animal Bite Treatment Center. Although PVRV usage rapidly increased between 2018 and 2021, PCEC remained limited until 2022, and ERIG administration remained consistently low.

**Fig. 6:**
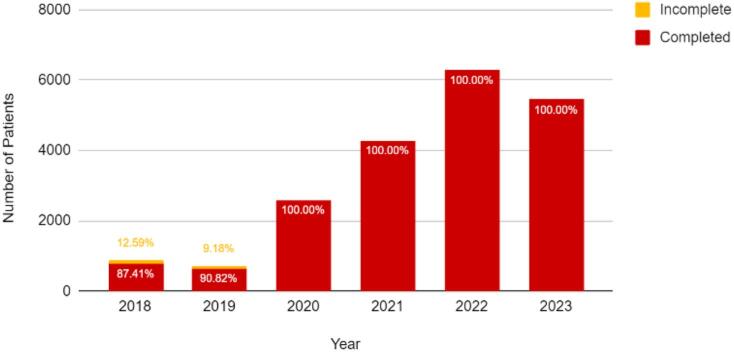
Number of complete and incomplete TCV vaccinations from 2018 to 2023.

Fig. 6 presents the annual rates of full and incomplete TCV coverage, with early challenges evident in 2018 when approximately 12.59 % (*n* = 111) of patients did not complete the vaccination series. These findings suggest gaps in both passive and active immunization adherence, particularly among high-risk patients, underscore the need for enhanced strategies to optimize PEP compliance and improve rabies prevention.

### 3.4 Factors affecting services offered by government-run animal bite treatment center

Table 3 shows the different parameters for the factors affecting rabies post-exposure prophylaxis services for this ABTC. There were six parameters: (1) service delivery and facilities, (2) health workforce, (3) health information systems, (4) access to essential medicines and facilities, (5) leadership, and (6) financing. Two of the six parameters were 100 % compliance, namely, the health workforce and leadership. Compliance in terms of financing parameters scored the lowest, followed by access to essential medicines and facilities, health information systems, and service delivery and facilities.

**Table 3:** Factors affecting the rabies post-exposure prophylaxis services of the government-run ABTC from 2018 to 2023.

Parameters	Results
I. Service delivery and facilities	12 out of 13 (92 %)
II. Health workforce	3 out of 3 (100 %)
III. Health information systems	10 out of 12 (83 %)
IV. Access to essential medicines and facilities (Technology)	5 out of 9 (56 %)
V. Leadership	3 out of 3 (100 %)
VI. Financing	1 out of 4 (25 %)

#### 3.4.1 Service delivery and facilities

The center successfully complied with the minimum requirements and implemented policies to maintain health, safety, and efficient services. The facilities were well maintained, featuring ample lighting, ventilation, pest control, and accessibility. Signages for directions, emergency evacuations, and adherence to smoking regulations are prominently displayed. The center adheres to an infection control policy for hygiene, antisepsis, and biohazard waste management. Only the referral system of the center was unable to comply with the minimum compliance requirements. Patient data were recorded on case record forms and classified using the NRPCP algorithm. Patient education was advocated through counseling, posters, and audio presentations. The center also actively participates in community activities, such as World Rabies Day and Rabies Awareness Month. In terms of vaccine delivery, vaccine schedules were closely monitored and patients with missed or delayed visits were contacted. The protocols were in place for dose adjustment based on delay length, with a new course required for significant delays. If vaccines were unavailable, the patients were referred to other ABTCs. Although existing protocols and referral forms were in place, they were not consistently practiced. Verbal referrals were sometimes used without proper documentation of patient follow-up or whether appropriate management was received for their animal bite cases.

#### 3.4.2 Health workforce

The center maintained a well-structured and skilled workforce with annual training. The center employed two physicians and 11 nurses, each with specific roles in diagnosis, vaccine administration, logistics, and management.

#### 3.4.3 Health information systems (HIS)

The center adhered to structured protocols for data collection, storage, and access, including vaccination card documentation, follow-ups, and routine updates. Quarterly and annual updates were conducted, although data inconsistencies and losses occurred because of frequent relocations and clerical errors. Additionally, the cohort analysis remained incomplete. Moreover, data were shared with national and local health authorities through the National Rabies Information System (NaRIS). However, intermittent technical issues can affect timely updates.

#### 3.4.4 Access to essential medicines and facilities

The center, certified by the Department of Health (DOH) to serve nine districts of Iloilo City until February 2025, offers both passive and active immunization for animal bites. It utilizes the Purified Verocell Rabies Vaccine (PVRV), purified chicken embryo vaccine (PCEC), and Equine Rabies Immunoglobulin (ERIG). The center maintains a detailed inventory of supplies and vaccines. It also has an infrastructure for cold chain management, with three vaccine refrigerators and backup generators, as well as daily temperature monitoring. However, the center faced challenges in terms of the supply chain, relying on the local government unit (LGU), and budget limitations resulted in a lack of HRIG supply. Additionally, the designated vaccine room was used as a record storage area, mixing files and personal supplies, which deviated from its intended function.

#### 3.4.5 Leadership

The center had a clear organizational structure with the designated roles outlined in its organizational chart. The District Health Officer oversees the center's operations, supervises the implementation of rabies policies, and coordinates with the DOH and local authorities for resources. Public nurses manage animal bite cases, maintain the rabies registry, provide health education, and handle defaulter tracing, whereas Rural Health Midwives assist in case management. Moreover, the policies were up-to-date, effectively communicated, and aligned with regulatory standards. Regular staff meetings and training programs were conducted.

#### 3.4.6 Financing

The center met only one health financing criterion: Philippine Health Insurance Corporation (PhilHealth) accreditation. As a government-operated facility, it relied heavily on the local government unit (LGU) for its operational budget, which covered the costs of rabies vaccines, HRIG/ERIG, and medications. The center operated under a request-based system, receiving supplies and operational support from the LGU, as needed. However, the adequacy of these supplies depends on LGU budget allocations, which may fluctuate. There were also no available records of the number of patients who received services via (1) ABTC-free vaccines, (2) PhilHealth Insurance subsidy, or (3) out-of-pocket expenditure.

## 4 Discussion

This study evaluated the performance and systemic constraints of a government-run, non-hospital-based Animal Bite Treatment Center (ABTC) in Iloilo City, Philippines, through the lens of the Andersen Health Service Utilization Model and the Penchansky and Thomas Framework [16,17]. These models situate service utilization within the dimensions of availability, accessibility, affordability, acceptability, and accommodation. From 2018 to 2023, the ABTC recorded 20,134 animal bite cases, with females (53.48 %) and adults aged >15 years (67.98 %) representing the majority of patients. Dog bites comprised 60.06 % of exposures, and most incidents were classified as Category II (72.12 %). Despite the geographic variability in case distribution, temporal analysis revealed no significant trends across demographic or exposure categories, indicating a stable risk of rabies within the population.

Demographic findings showing higher cases among females and adults align with the behavioral determinants outlined in the Andersen model, suggesting persistent sex- and age-specific disparities in health-seeking behaviors [3,6,9,11]. Addressing these socio-behavioral differences through tailored communication campaigns targeting males and children is essential to ensure timely consultation and equitable PEP access [18,19]. Beyond these demographic variations, service utilization patterns and vaccine adherence outcomes further illustrate ABTC's operational capacity and responsiveness to the population's needs.

Trend analysis from 2018 to 2023 revealed no significant change in animal bite incidence, indicating limited progress in upstream prevention programs such as dog vaccination, pet population control, and enforcement of the Anti-Rabies Act. The persistently stable exposure rate suggests that although ABTC operations effectively meet case management needs, community-level preventive interventions have not sufficiently reduced the bite incidence. Improvements in TCV completion rates, such as the initial 12.59 % incomplete vaccination rate in 2018, declined markedly in later years, demonstrating enhanced patient monitoring and follow-up. Nonetheless, incomplete reporting to the National Rabies Information System (NaRIS) and clerical inconsistencies in data recording continue to limit forecasting accuracy and efficient resource allocation [5].

Within this framework, the ABTC exhibited strong acceptability, accessibility, and accommodation, as evidenced by the uninterrupted vaccine administration during the COVID-19 pandemic and the high completion rates for TCV regimens [1,5,[18], [19], [20]]. These strengths highlight community trust, dependable service continuity, and competent local leadership, demonstrating that essential facility-level functions remain robust, even under resource-constrained conditions. However, the supply instability remains a critical constraint. The sharp decline in ERIG administration, limited to only 42.65 % of Category III patients in 2023, highlights weaknesses in supply chain governance and financing. As ERIG is vital for severe exposure, such shortages increase the risk of preventable fatalities, consistent with the observed increase in rabies-related deaths between 2021 and 2023. These deficiencies reflect reliance on unpredictable vaccine procurement systems and variable local government budgets, perpetuating inequities in availability and affordability despite sustained demand [21,22].

The number of rabies-related mortalities recorded at the ABTC remained relatively low, likely reflecting the center's high completion rates for post-exposure vaccination and improved treatment adherence within the community. However, the gradual increase in fatalities observed toward 2023 coincided with a reduction in RIG administration among high-risk patients. This suggests that, while ARV coverage has been effectively sustained, incomplete RIG delivery may have contributed to residual fatal outcomes, particularly in individuals with Category III exposure. This pattern aligns with the findings of Dimaano et al. and Gongal et al., who noted that limited RIG availability and incomplete PEP adherence remained major contributors to rabies mortality in the Philippines and other Asian countries. Similarly, the Iloilo Provincial Health Office reported a province-wide increase in rabies cases, accompanied by interruptions in vaccine supply. Therefore, ensuring a stable and affordable supply of RIG is crucial for sustaining low mortality rates and preventing further increases in mortality.

Viewed through the WHO Health System Building Blocks Framework [23], ABTC demonstrates strong performance in service delivery, workforce capacity, and managerial leadership but continues to face deficiencies in financing, supply chain resilience, and information management systems. These findings highlight the interdependence of facility-level effectiveness and systemic enablers. The stable incidence of bites further highlights the need to scale up preventive strategies, strengthen data-driven planning, and integrate rabies control initiatives across the health, veterinary, and community sectors [3,24].

Anchored in the Andersen and WHO frameworks, these systemic findings inform targeted policy reforms necessary to ensure future program sustainability. Policy implications point toward institutionalizing integrated multilevel support mechanisms grounded in global best practices. Strengthening vaccine supply chain logistics should be prioritized by establishing a centralized digital inventory system linked to the National Rabies Information System (NaRIS) and supported by regional buffer stocks to minimize delays and ensure equitable access to both anti-rabies vaccines (ARV) and rabies immunoglobulin (RIG) [20]. Forecast-based budgeting and continuous data validation would enhance coordination between the Department of Health and local governments. Capacity-building programs on digital literacy, data management, and logistics coordination for ABTC personnel can further improve the accuracy and timeliness of supply management, as evidenced by digital health initiatives in low-income and middle-income countries [25]. These priorities align with lessons from the COVID-19 pandemic, which highlighted that real-time data systems, synchronized logistics, and a skilled health workforce are as critical as infrastructure in maintaining an uninterrupted vaccine delivery [26]. Sustained public education campaigns should reinforce responsible pet ownership, early consultations after animal bites, and adherence to PEP regimens.

Overall, Iloilo City's non-hospital-based government-run ABTC provides an empirical model for how local health systems can operate effectively under decentralized governance while addressing systemic constraints. Interpreted through the Andersen Behavioral Model and the WHO Health Systems Framework, the findings demonstrate that achieving universal access to rabies post-exposure prophylaxis requires aligning governance, logistics, and behavioral factors into an integrated framework. Strengthening vaccine supply chain management, reinforcing digital health information systems, investing in the health workforce, and institutionalizing community partnerships are critical steps toward achieving the national vision of a rabies-free Philippines by 2030.

### 4.1 Study limitations

The data derived from this study relied on the completeness and accuracy of the data management practices at the center. The statistical insights derived may not fully represent the rabies situation in the entirety of Iloilo City, as they reflect the operations of only one of the two government-run Animal Bite Treatment Centers (ABTCs) and did not account for services provided by private Animal Bite Centers (ABCs). Furthermore, data collection was confined to the observations and protocols specific to this center, which may have introduced a potential bias based on the operational procedures, internal policies, and practices currently in place. Consequently, any aspects of service delivery that were not explicitly captured by these established procedures were not reflected in the study results, potentially affecting their comprehensiveness and accuracy.

## CRediT authorship contribution statement

**Anna-Lee B. Bandoy:** Writing – original draft, Visualization, Validation, Methodology, Investigation, Formal analysis, Data curation, Conceptualization. **Neil Marc S. Dasas:** Writing – original draft, Visualization, Validation, Methodology, Investigation, Formal analysis, Data curation, Conceptualization. **Alpha Issa Christianne P. Abegonia:** Writing – original draft, Methodology, Investigation, Formal analysis, Data curation. **John Piox J. Badiang:** Writing – original draft, Validation, Methodology, Investigation, Formal analysis, Data curation. **Chester Lloyd S. Berdan:** Writing – original draft, Validation, Methodology, Investigation, Formal analysis. **Maria Lucille M. Magallanes:** Writing – original draft, Methodology, Investigation, Formal analysis. **Jes Ivan D. Sian:** Writing – original draft, Methodology, Investigation, Formal analysis, Data curation. **Therese A. Sumague:** Writing – original draft, Validation, Methodology, Investigation, Formal analysis, Data curation. **Patricia Kei P. Tulio:** Writing – original draft, Methodology, Investigation, Formal analysis, Data curation. **Addy Mae P. Binoya:** Writing – original draft, Methodology, Investigation, Formal analysis, Data curation. **Ma. Cristina L. Erum:** Writing – original draft, Methodology, Investigation, Formal analysis, Data curation. **Alfredo A. Hinay:** Writing – review & editing, Writing – original draft, Supervision, Project administration.

## Declaration of competing interest

The authors declare no conflict of interest. All authors have approved this manuscript, agree with its submission, and share collective responsibility and accountability. This manuscript has not been published or is not under review by another journal.
